# On the speaker discriminatory power asymmetry regarding acoustic-phonetic parameters and the impact of speaking style

**DOI:** 10.3389/fpsyg.2023.1101187

**Published:** 2023-04-17

**Authors:** Julio Cesar Cavalcanti, Anders Eriksson, Plinio A. Barbosa

**Affiliations:** ^1^Laboratory of Phonetics, Department of Linguistics, Stockholm University, Stockholm, Sweden; ^2^Institute of Language Studies, Department of Linguistics, University of Campinas, Campinas, Brazil

**Keywords:** speech analysis, phonetics, acoustic phonetics, forensic phonetics, speaker comparison

## Abstract

This study aimed to assess what we refer to as *the speaker discriminatory power asymmetry* and its forensic implications in comparisons performed in different speaking styles: spontaneous dialogues vs. interviews. We also addressed the impact of data sampling on the speaker's discriminatory performance concerning different acoustic-phonetic estimates. The participants were 20 male speakers, Brazilian Portuguese speakers from the same dialectal area. The speech material consisted of spontaneous telephone conversations between familiar individuals, and interviews conducted between each individual participant and the researcher. Nine acoustic-phonetic parameters were chosen for the comparisons, spanning from temporal and melodic to spectral acoustic-phonetic estimates. Ultimately, an analysis based on the combination of different parameters was also conducted. Two speaker discriminatory metrics were examined: Cost Log-likelihood-ratio (*Cllr*) and Equal Error Rate (*EER*) values. A general speaker discriminatory trend was suggested when assessing the parameters individually. Parameters pertaining to the temporal acoustic-phonetic class depicted the weakest performance in terms of speaker contrasting power as evidenced by the relatively higher *Cllr* and *EER* values. Moreover, from the set of acoustic parameters assessed, spectral parameters, mainly high formant frequencies, i.e., F3 and F4, were the best performing in terms of speaker discrimination, depicting the lowest *EER* and *Cllr* scores. The results appear to suggest a speaker discriminatory power *asymmetry* concerning parameters from different acoustic-phonetic classes, in which temporal parameters tended to present a lower discriminatory power. The speaking style mismatch also seemed to considerably impact the speaker comparison task, by undermining the overall discriminatory performance. A statistical model based on the combination of different acoustic-phonetic estimates was found to perform best in this case. Finally, *data sampling* has proven to be of crucial relevance for the reliability of discriminatory power assessment.

## 1. Introduction

Signal-based analyses of melodic, spectral, and temporal acoustic-phonetic parameters are common procedures within forensic speaker comparison practice, allowing the overall description of the differences between individuals' *manner of speaking*. In this context, when conducting an international survey on forensic speaker comparison practices with 36 participants from 13 countries, Gold and French (2011) noted that among those forensic experts conducting some kind of phonetic acoustic analysis, 100% reported analyzing some of the fundamental frequency (melodic) descriptors, 97% carried out some form of formant examination on vowels (spectral), and 81% reported conducting a formal measure of speech tempo (temporal) in their practices.

However, it is necessary to take into account that, just as it applies to the comparison of parameters within the same phonetic scope, an homogeneous level of speaker discriminatory power should not be presumed when estimates deriving from different acoustic-phonetic classes are appraised. Previous research - yet, very few- has acknowledged some parameters' relatively poorer discriminatory potential in relation to others from a distinct phonetic class, cf. Künzel (1997), Hughes et al. (2013), and Lennon et al. (2019). Most other studies on the impact of speaking style on acoustic-phonetic parameters are mainly concerned with linguistic rather than forensic implications. Moreover, they use methodological designs different from those used in forensic research.

Concerning the desired properties of a candidate parameter for the forensic speaker discriminatory application, two attributes– among others– have been suggested, namely, a low intra-speaker variability and a high inter-speaker variability, cf. Nolan (1983). These properties are reflected in the discriminatory power estimates assessed in the present study.

In a study examining speech timing parameters, such as speech and articulation rates, to determine their discriminatory power in German, Künzel (1997) found that, based on equal error distributions, the ability of these measures to contrast speakers was relatively poor. As pointed out by the author, this appears particularly notable when comparing temporal estimates with other acoustic parameters, such as those based on linear predictive coding (LPC) or cepstral coefficients. Equal error rate (*EER*) values for speech and articulation rates in three speaking conditions were roughly 50 and 38%, respectively—note that, for *EER*, the closer to 50%, the lower the discriminatory performance of a given parameter is. However, the author emphasized that, unlike most acoustic parameters, an estimate such as articulation rate is more appropriate for use under real-world forensic conditions, often involving telephone transmitted speech. Speech temporal parameters tend to be more resistant to poor sound quality and different sources of audio degradation (e.g., external noise).

An experiment conducted by Lennon et al. (2019) with 30 English speakers aimed to compare common speaking rate measures based on the counting of canonical and surface syllables, phones, and CV segments. It was demonstrated that these rates were closely inter-correlated, yielding similar discriminating powers. However, as remarked by the researchers, the results suggested that “*tempo*” is a relatively poor speaker discriminant regardless of the methodological choices made, being characterized by rather high *EERs* and Cost log-likelihood-ratio (*Cllr*) values close to 1. *EER* values tend to vary from 0 to 0.50, and *Cllr* from 0 to 1, where the closer to 0 the best the discriminatory performance is for both estimates. In Lennon et al. (2019), *EER* values varied from 0.28 to 0.37. As for *Cllr* values, these varied from 0.88 to 0.89.

The same tendency was revealed in the study carried out by Hughes et al. (2013) with English-speaking subjects on the implication of reference sample size and the calculation of numerical likelihood ratio (LR) based on articulation rate. In that study, both *EER* and *Cllr* average values were relatively high 0.35 and 0.97, respectively, suggesting an overall poor performance of articulation rate for forensic speaker comparisons. Furthermore, it was observed that the *EER* estimate tended to remain stable/consistent with the increase in the number of tokens, not presenting important repercussions in terms of system validity. As for *Cllr*, calibrated LRs were found to be robust to sample size effects, whilst non-calibrated scores displayed much more sensitivity to the amount of reference data used.

Given the necessity of better understanding such a discriminatory power *asymmetry* regarding parameters deriving from different acoustic-phonetic classes, the present study aims to assess the speaker-discriminating potential of some of the estimates commonly regarded as the most discriminatory within their phonetic domain (i.e., temporal, spectral, and melodic). Furthermore, a new language (Brazilian Portuguese) was assessed, which further justifies the present analysis. Knowing what to expect in terms of discriminatory power performance from different classes of parameters is of great relevance for the forensic speaker comparison practice, making it possible to weigh their potentials and limitations.

The methodological design represents an advance on previous work by applying a more systematic approach: the analysis of the same speakers and the same stretches of speech material when assessing parameters deriving from different acoustic-phonetic classes. Furthermore, the impact of *data sampling* on the discriminatory performance of the parameters is also considered.

Assessing the *asymmetry* regarding the speaker-discriminating power of different acoustic measures may be relevant both from a practical and theoretical viewpoint, shedding light on the very nature of speech variability from an acoustic-based perspective and the limitations of their usage in speaker comparison tasks.

## 2. Materials and methods

The present study registered under the protocol 95127418.7.0000.8142 was evaluated and approved by the ethical committee at Campinas State University (UNICAMP). All participants voluntarily agreed to be part of the research verbally and by signing a participant consent form.

### 2.1. Participants

The participants were 20 speakers, ten identical male twin pairs, Brazilian Portuguese (BP) speakers from the same dialectal area. The participants' age ranged between 19 and 35 years, with a mean of 26.4 years.

Although the speakers in the present study were part of a twin study, in which identical twin pairs were systematically analyzed regarding possible acoustic-phonetic differences in their productions, the present study focusses on comparisons among all participants in the corpus (cross-pair comparisons).

However, it should be noted that the presence of twin pairs in the dataset (roughly 5% of the total speaker comparison number) adds a higher level of difficulty in the speaker discrimination task, making the speaker separation a bit more challenging. This higher level of difficulty is very welcome when considering that only high-quality recordings were used, which may result in more optimistic outcomes.

### 2.2. Recordings

All recordings were made with a sample rate of 44.1 kHz and 16-bit, using an external audio card (Focusrite Scarlett 2i2) and two headset condenser microphones (DPA 4066-B). The recordings were undertaken in silent rooms located in the cities where the speakers resided. The recordings were made in two different sessions, as described below. Approximately, 5–10 min of unedited conversational speech (dialogue) and 3–5 mins of unedited interview speech were available per speaker.

#### 2.2.1. Session I

The speech materials recorded in session I consisted of spontaneous telephone conversations between very familiar subjects (twin brothers), with dialog topics being decided by the pairs. During this recording session, the speakers were placed in different rooms, not seeing each other, and only hearing each other via mobile phones. The speakers were encouraged to start a conversation using a mobile phone while being simultaneously recorded by high-quality microphones. The unedited and unfiltered audio signals were then processed and registered in two separate channels, with all their acoustic properties preserved.

#### 2.2.2. Session II

In recording session 2, speakers were interviewed by the researcher. At this stage, the participants were asked to describe their everyday routine from the moment they wake up until they go to bed. Moreover, they were asked to describe what they usually do in their free time.

After they had described what their overall routine looked like, the same question was extended to previous weeks, in which the participants were asked to describe what their routine looked like a week, a month, and a year ago. They were also questioned about how their routines changed in the course of the year.

Apart from the fact that a different speaking style was elicited by the interviewer, an important aspect in this step regards the reduction in the level of familiarity between the interlocutors. Such a reduction was intended and aimed to reproduce what often takes place in a forensic speaker comparison setting, where individuals are interrogated by an interviewer with whom they are not familiar.

### 2.3. Data segmentation and transcription

All speech material was segmented and transcribed manually in the Praat software (Boersma and Weenink, 2018), following acoustic (i.e., the analysis of spectrogram traces) and auditory criteria. Data annotation, which is relevant for the present analysis, comprised five distinct textgrid tiers, as follows:

- Dialogue/interview parts: different portions of the dialogues/interviews throughout the recordings, e.g., beginning, middle, and final parts;
- Speech chunks: speech intervals on average 3 s long, in most cases corresponding to inter-pause intervals (i.e., stretches of speech between longer pauses);
- Vowel-to-vowel intervals: syllable-sized units defined as all the segments uttered between two consecutive vowel onsets;
- Oral monophthongs: oral monophthongs contained within the speech chunks;
- Silent pauses: silent pauses with a minimum duration threshold of 100 ms.

### 2.4. Acoustic-phonetic parameters

Overall, nine parameters deriving from three different acoustic-phonetic classes, i.e., melodic, spectral, and temporal, were assessed as follows:

- ***f*****0 median**: *f* 0 median in semitones ref 1 Hz and in Hertz;
- ***f*****0 base value**: base value of *f* 0 in semitones ref 1 Hz and in Hertz (i.e., equivalent to the 7.64th quantile of the *f* 0 sample);
- **F1**: the first formant frequency in Hertz measured at oral vowels mid-points;
- **F2**: the second formant frequency in Hertz measured at oral vowels mid-points;
- **F3**: the third formant frequency in Hertz measured at oral vowels mid-points;
- **F4**: the fourth formant frequency in Hertz measured at oral vowels mid-points;
- **Silent pauses duration**: defined as the duration of final and non-final silent pauses with a minimum duration of 100 ms;
- **Vowel duration**: defined as the duration of oral monophthongs including the seven phonemic vowels of Brazilian Portuguese in all possible contexts of realization that emerged;
- **V-V units duration**: vowel-to-vowel intervals, which are syllable-sized units defined as all the segments uttered between two consecutive vowel onsets.

Scripts developed specifically for the Praat software were used for the parameters' extraction, cf. Barbosa (2015, 2020). For the automated *f* 0 extraction, the floor and ceiling were defined as 60–300 Hz. *f* 0 estimates were extracted from connected speech intervals (speech chunks on average 3 s long).

A decision was made to perform an analysis that is mainly operated on the same kind of unit, namely the vowel. The only two exceptions here are the silent pauses and V-V unit duration parameters. However, when testing the combined discriminatory power of different acoustic-phonetic parameters, only vowel segments were chosen.

Vowels in all prosodic contexts were considered for the formant extractions, and were assessed regarding their duration patterns. These were grouped according to their different qualities, as best described further.

Note that, the assessment of centrality fundamental frequency parameters, formant frequency extractions, and durations represent typical choices of parameters used in forensic speaker comparisons, cf. Gold and French (2011).

### 2.5. Statistical analysis

Regarding the assessment of speaker discriminatory power of spectral, melodic, and speech timing acoustic-phonetic parameters, two estimates were examined as a function of the comparisons among all speakers in the study using the script “fvclrr” (Lo, 2020) in the software R for statistical analyses (R Core et al., 2021).

The first estimate is the Cost Log-likelihood-ratio function (Cllr), an empirical estimate of the precision of likelihood ratios proposed by Brümmer and Du Preez (2006) and applied, among others, by Morrison (2009). For computing such an estimate, likelihood ratios were calculated through Multivariate Kernel Density analysis—MVKD (Aitken and Lucy, 2004), which is a non-parametric approach. Multiple pairwise comparisons were performed across individuals in which the background sample consisted of data from all speakers, except those being directly compared (i.e., cross-validation).

Parameters depicting *Cllr* values lower than 1 point to some level of speaker specificity and potential relevance for the forensic speaker comparison application; whereas *Cllr* values around or above 1 suggest an overall poor discriminatory performance. As such, the closer to 0 the *Cllr* value is, the better the parameter's discriminatory power is.

The second estimate is the Equal Error Rate (*EER*), which represents the point where the false rejection rate (type I error) and false acceptance rate (type II error) are equal, as is used to describe the overall accuracy of biometric systems (Conrad et al., 2012). This estimate was generated along with the *Cllr*. Lower *EER* values are compatible with better accuracy, whereas higher *EER* values suggest worse discriminatory performance. In the present study, both *Cllr* and *EER* values were reported after performing several tests with the randomly selected data points. Such a procedure is described more fully in the following subsection of the paper (i.e., the downsampling procedure).

Fusion and calibration procedures were employed when combining different parameters to assess their joint discriminatory capacity. Such procedures result in the combination of likelihood ratio (LR) scores from (multiple) test systems to provide a single set of fused LR scores based on a logistic regression model trained with the same set of data (i.e., self-calibration). Such a process is exploited in Morrison et al. (2011) and is regarded as an adequate solution when combining multiple estimates of likelihood ratios on the same data, cf. Morrison et al. (2019), such as the combined discriminatory power of different vowels.

For the calculation of LR's within the same speaking style (i.e., dialogue and interview), a cross-validation procedure was adopted, in which multiple pairwise comparisons were performed across subjects with the background sample consisting of data from all speakers, except those being directly compared.

For the calculation of LR's in speaker comparison in mismatched speaking styles (i.e., interview vs. dialogue), the interview data was treated as the reference material and the dialogue data was treated as the questioned material. At this stage, multiple downsamplings were also performed to keep the number of observations for each speaking style constant.

LR scores of single parameters presented in this study were also calibrated to reduce the magnitude and incidence of likelihood ratios known to support the incorrect hypothesis, i.e., the contrary-to-fact hypothesis, thereby improving accuracy, cf. Morrison et al. (2011, 2019). As a result, all *Cllr* values reported here were calibrated.

### 2.6. Downsampling procedure

Given the nature of the speech material analyzed, i.e., spontaneous dialogues, a discrepancy regarding the number of samples produced per speaker and dyad was observed. Furthermore, as can be expected, some speakers tend to hold their conversation turns for a more extended period resulting in the emergence of a higher number of tokens and, consequently, imbalanced data across different speakers. In view of that, a resampling/downsampling procedure was conducted to ensure that all speakers were represented by the same number of data points for all tested parameters.

The resampling/downsampling procedure applied here consisted of randomly sampling the data set so that all classes were required to have the same frequency of occurrence as the minority case. In this case, the speaker with the lowest number of speech chunks or vowel tokens was considered the minority case. Moreover, since the data selection for the downsampling (from a larger dataset) was random, it was repeated 200 times to minimize the selection bias in the calculation of the cumulative *Cllr* and *EER*'s median, minima, and maxima. For this procedure, the R package “recipes” (Kuhn and Wickham, 2020) was employed.

*Cllr* and *EER* values were reported after performing 200 tests with the randomly selected data points for each individual or combination of acoustic-phonetic parameters. As a result, variability curves from the performance of the parameters assessed could be visualized as a function of data resamplings throughout each independent replication. This provides relevant information on the system stability. Average, minimum, maximum *Cllr* and *EER* values were then computed.

The number of data points for each type of speech unit is displayed in Table 1. For tests carried out with the spontaneous dialogue material, from a data set comprising 9,960 oral vowels, a subset of 8,640 vowel data points was randomly selected and analyzed, namely, 432 vowels per speaker in each of the 200 resamplings/downsamplings. Moreover, from a total of 11,736 V-V units (including V-V units with oral and nasalized vowels), 7,860 V-V units were randomly selected and analyzed in each of the 200 replications. As for silent pauses, from 853 available silent pause data points, 440 were randomly selected and analyzed.

**Table 1:** Number of data points for each speech unit analyzed: total and downsampled numbers for each speaking style.

**Speech units**	**Total**	**Dialogue**	**Interview**	**Dialogue downsampled**	**Interview downsampled**
Vowels	16,352	9,960	6,905	8,640 (432 per speaker)	5,240 (262 per speaker)
V-V units	21,242	11,736	9,506	7,860 (393 per speaker)	7,240 (362 per speaker)
Silent pauses	1,748	853	895	440 (22 per speaker)	600 (30 per speaker)

For the interview speech material, from a data set comprising 6,905 oral vowels, 5,240 vowel data points were randomly selected for the analyses. From 9,506 V-V units (including V-V units with oral and nasalized vowels), 7,240 were randomly selected and analyzed. Finally, for silent pauses, from 895 available silent pause data points, 600 were randomly selected and analyzed.

Finally, for the assessment of the combined discriminatory power of different acoustic-phonetic estimates in the mismatched speaking style comparison condition (spontaneous dialogue vs. interview), multiple downsamplings were also performed to keep the number of observations for each speaking style the same.

## 3. Results

The results regarding the assessment of individual parameters for each testing condition are summarized in Table 2. As for the combination of different acoustic-phonetic parameters, the results are provided in Table 3.

**Table 2:** *Cllr* and *EER* values as a function of testing condition (dialogue, interview, dialogue vs. interview) for each individual acoustic-phonetic parameter.

***Cllr***									
**Dialogue**									
	Silent p. dur	Vowel dur	VV dur	f0 median	f0 base value	F1	F2	F3	F4
Average	0.96	0.81	0.83	0.39	0.38	0.29	0.47	0.25	0.29
Minima	0.86	0.62	0.69	0.31	0.37	0.19	0.32	0.10	0.20
Maxima	1.00	0.94	0.94	0.51	0.39	0.42	0.63	0.42	0.42
SD	0.03	0.05	0.05	0.04	0.00	0.05	0.06	0.05	0.05
**Interview**									
Average	0.90	0.82	0.82	0.45	0.50	0.38	0.57	0.34	0.38
Minima	0.79	0.69	0.67	0.33	0.49	0.25	0.33	0.22	0.20
Maxima	0.99	0.94	0.92	0.61	0.52	0.54	0.75	0.47	0.58
SD	0.04	0.05	0.05	0.04	0.00	0.05	0.07	0.05	0.06
**Dialogue vs. interview**									
Average	1.00	0.87	0.95	0.67	0.67	0.66	0.67	0.57	0.60
Minima	1.00	0.68	0.91	0.57	0.57	0.59	0.53	0.45	0.47
Maxima	1.00	0.96	0.97	0.75	0.75	0.76	0.80	0.66	0.71
SD	0.00	0.05	0.01	0.03	0.03	0.03	0.05	0.04	0.05
***EER***									
**Dialogue**									
	Silent p. dur	Vowel dur	VV dur	f0 median	f0 base value	F1	F2	F3	F4
Average	0.42	0.31	0.34	0.12	0.14	0.08	0.15	0.07	0.09
Minima	0.30	0.20	0.25	0.07	0.11	0.04	0.09	0.01	0.04
Maxima	0.60	0.45	0.45	0.18	0.15	0.14	0.24	0.14	0.15
SD	0.05	0.04	0.04	0.02	0.01	0.02	0.03	0.02	0.02
**Interview**									
Average	0.37	0.32	0.33	0.16	0.15	0.11	0.19	0.11	0.12
Minima	0.25	0.20	0.25	0.10	0.15	0.05	0.10	0.05	0.05
Maxima	0.46	0.44	0.46	0.20	0.19	0.20	0.35	0.20	0.20
SD	0.04	0.04	0.04	0.02	0.01	0.03	0.04	0.03	0.03
**Dialogue vs. interview**									
Average	0.50	0.35	0.38	0.28	0.27	0.26	0.25	0.20	0.19
Minima	0.45	0.24	0.31	0.20	0.18	0.19	0.15	0.12	0.14
Maxima	0.55	0.50	0.45	0.35	0.35	0.34	0.34	0.28	0.26
SD	0.01	0.05	0.03	0.03	0.03	0.03	0.04	0.03	0.03

**Table 3:** *Cllr* and *EER* values as a function of combination of acoustic-phonetic parameters in mismatched speaking style comparisons (dialogue vs. interview).

**Dialogue vs. interview**				
***Cllr***				
	**Average**	**Minima**	**Maxima**	**SD**
f0 median + f0 base value	0.62	0.48	0.71	0.03
f0 median + Vowel dur	0.57	0.44	0.68	0.04
f0 base value + Vowel dur	0.56	0.43	0.68	0.04
f0 median + f0 base value + Vowel dur	0.52	0.38	0.67	0.05
F1 + F2	0.47	0.34	0.57	0.04
F1 + F2 + Vowel dur	0.39	0.24	0.50	0.05
F1 + F2 + f0 median	0.33	0.23	0.44	0.04
F1 + F2 + f0 base value	0.33	0.20	0.45	0.04
F1 + F2 + F3	0.31	0.17	0.40	0.05
F3 + F4	0.30	0.21	0.40	0.04
F3 + F4 + Vowel dur	0.23	0.15	0.34	0.04
F3 + F4 + f0 median	0.14	0.00	0.24	0.04
F3 + F4 + f0 base value	0.14	0.00	0.25	0.04
F1 + F2 + F3 + f0 base value + Vowel dur	0.11	<0.01	0.26	0.07
F3 + F4 + f0 base value + Vowel dur	0.07	<0.01	0.24	0.06
F1 + F2 + F3 + f0 median + f0 base value + Vowel dur	0.03	<0.01	0.19	0.05
F1 + F3 + F4 + f0 base value + Vowel dur	<0.01	<0.01	0.13	0.02
***EER***				
	**Average**	**Minima**	**Maxima**	**SD**
f0 median + f0 base value	0.24	0.16	0.30	0.02
f0 median + Vowel dur	0.20	0.10	0.27	0.03
f0 base value + Vowel dur	0.19	0.11	0.26	0.03
f0 median + f0 base value + Vowel dur	0.17	0.10	0.25	0.03
F1 + F2	0.15	0.09	0.21	0.03
F1 + F2 + Vowel dur	0.12	0.05	0.19	0.03
F1 + F2 + f0 median	0.08	0.04	0.14	0.02
F1 + F2 + f0 base value	0.09	0.05	0.14	0.02
F1 + F2 + F3	0.08	0.02	0.15	0.02
F3 + F4	0.09	0.05	0.15	0.02
F3 + F4 + Vowel dur	0.06	0.01	0.14	0.02
F3 + F4 + f0 median	0.03	0.00	0.06	0.02
F3 + F4 + f0 base value	0.03	0.00	0.06	0.02
F1 + F2 + F3 + f0 base value + Vowel dur	0.01	0.00	0.05	0.01
F3 + F4 + f0 base value + Vowel dur	<0.01	0.00	0.05	0.01
F1 + F2 + F3 + f0 median + f0 base value + Vowel dur	<0.01	0.00	0.04	0.01
F1 + F3 + F4 + f0 base value + Vowel dur	<0.01	0.00	0.03	<0.01

**Figure 1:**
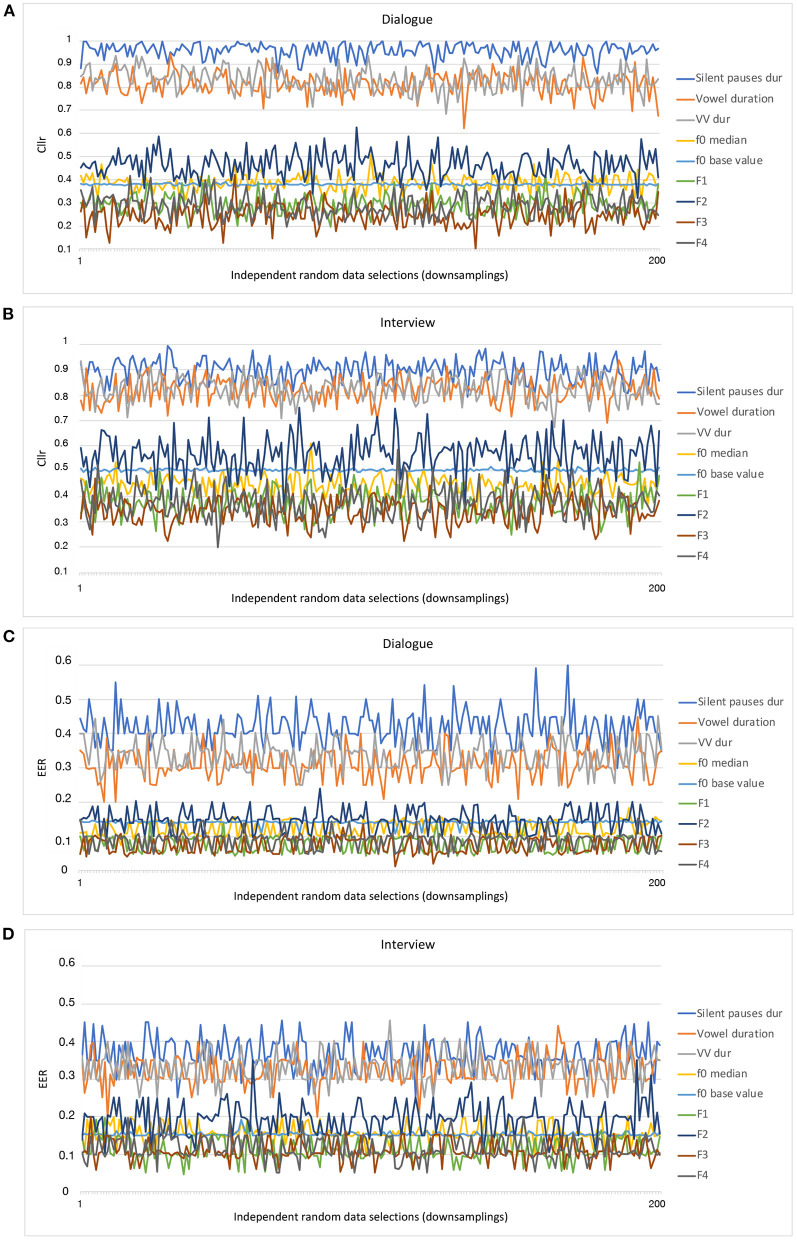
*Cllr* **(A, B)** and *EER* **(C, D)** curves as a function of data sampling (downsamplings) for different acoustic-phonetic parameters and speaking styles.

In Figure 1, the evolution of *Cllr* and *EER* values across several downsampling replications can be visually inspected as a function of individual parameters. The *x*-axis depicts different resampling/downsampling of the larger data (up to 200), whereas the *y*-axis depicts the Cllr and EER range. It should be noted that each individual resampling/downsampling of the larger data (in the *x*-axis) was independent of each other, in the sense that for each time a downsampling was conducted, a new data configuration is obtained. As such, those curves are the expression of what can be regarded as a “selection bias” and the uncertainty regarding the discriminatory performance given different data configurations.

**Figure 2:**
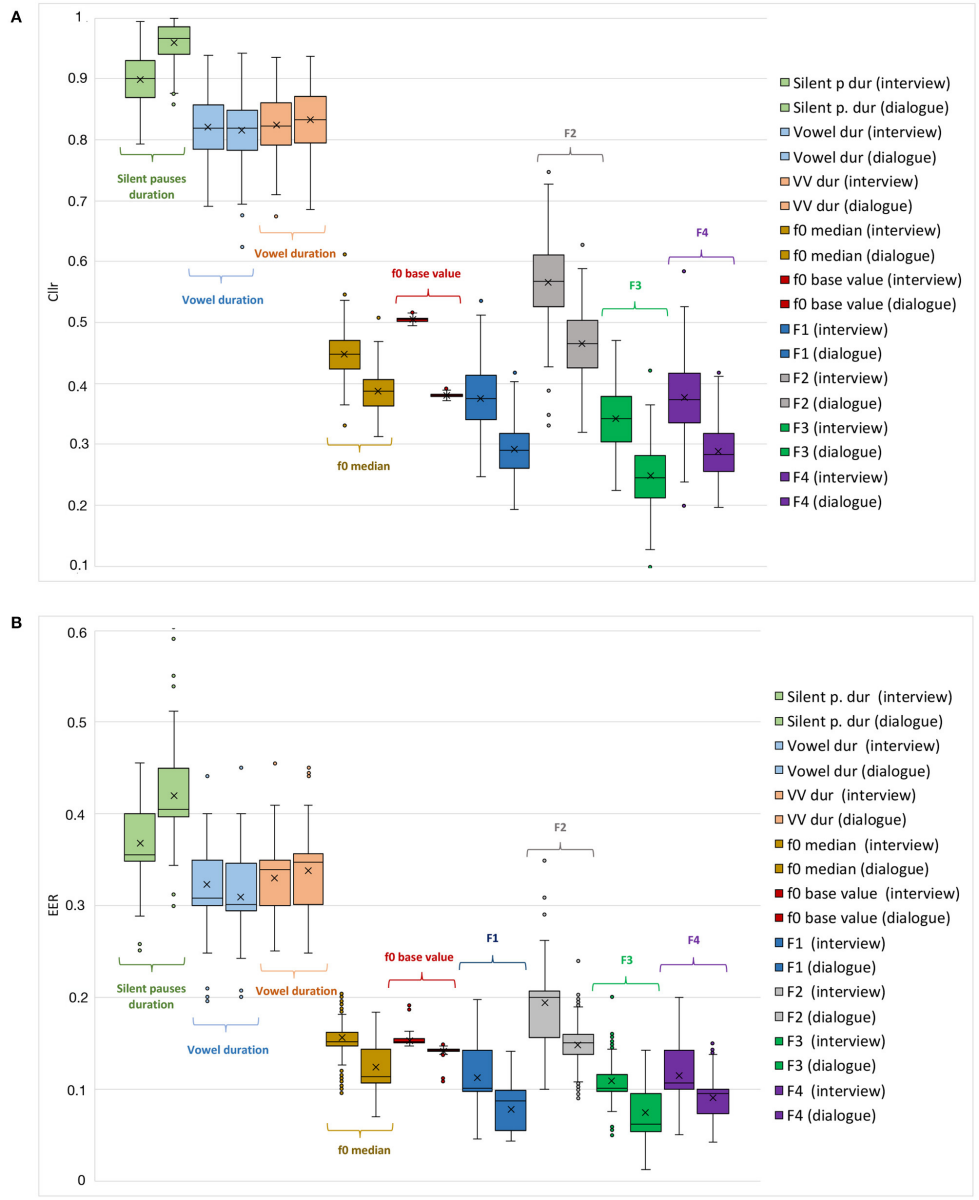
*Cllr* **(A)** and *EER* **(B)** cumulative values as a function of different acoustic-phonetic parameters and speaking styles, i.e., interview (left boxes), and dialogue (right boxes).

In Figure 2, general trends regarding the performance of such parameters can be visualized in the form of boxplots, in which cumulative values are depicted for each speaking style (i.e., dialogue and interview).

**Figure 3:**
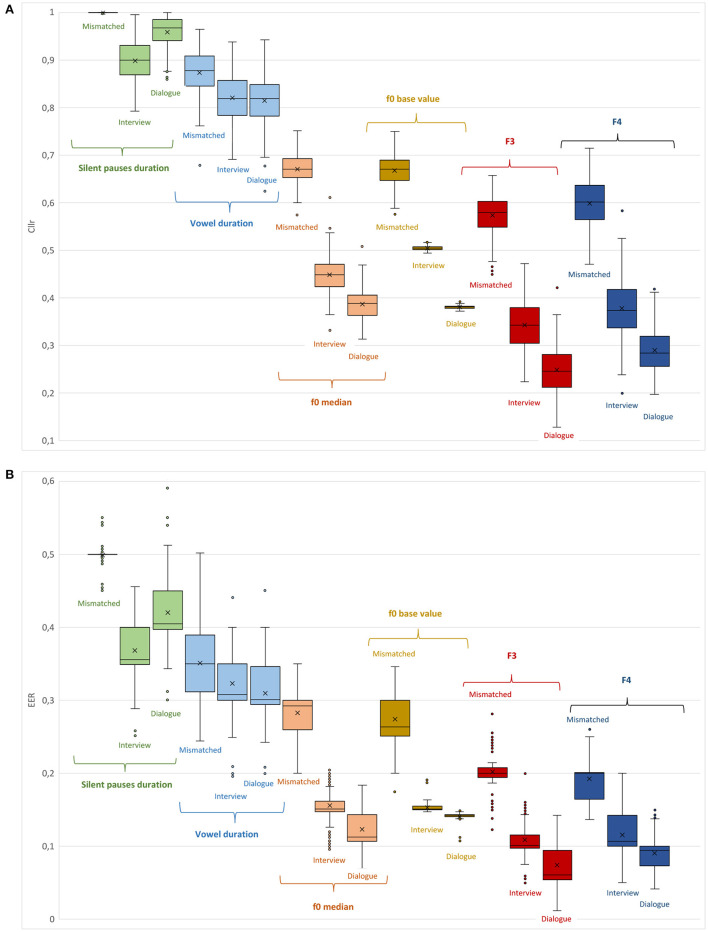
*Cllr* **(A)** and *EER* **(B)** cumulative values as a function of different acoustic-phonetic parameters and speaking styles, i.e., interview, dialogue, and interview vs. dialogue (mismatched).

**Figure 4:**
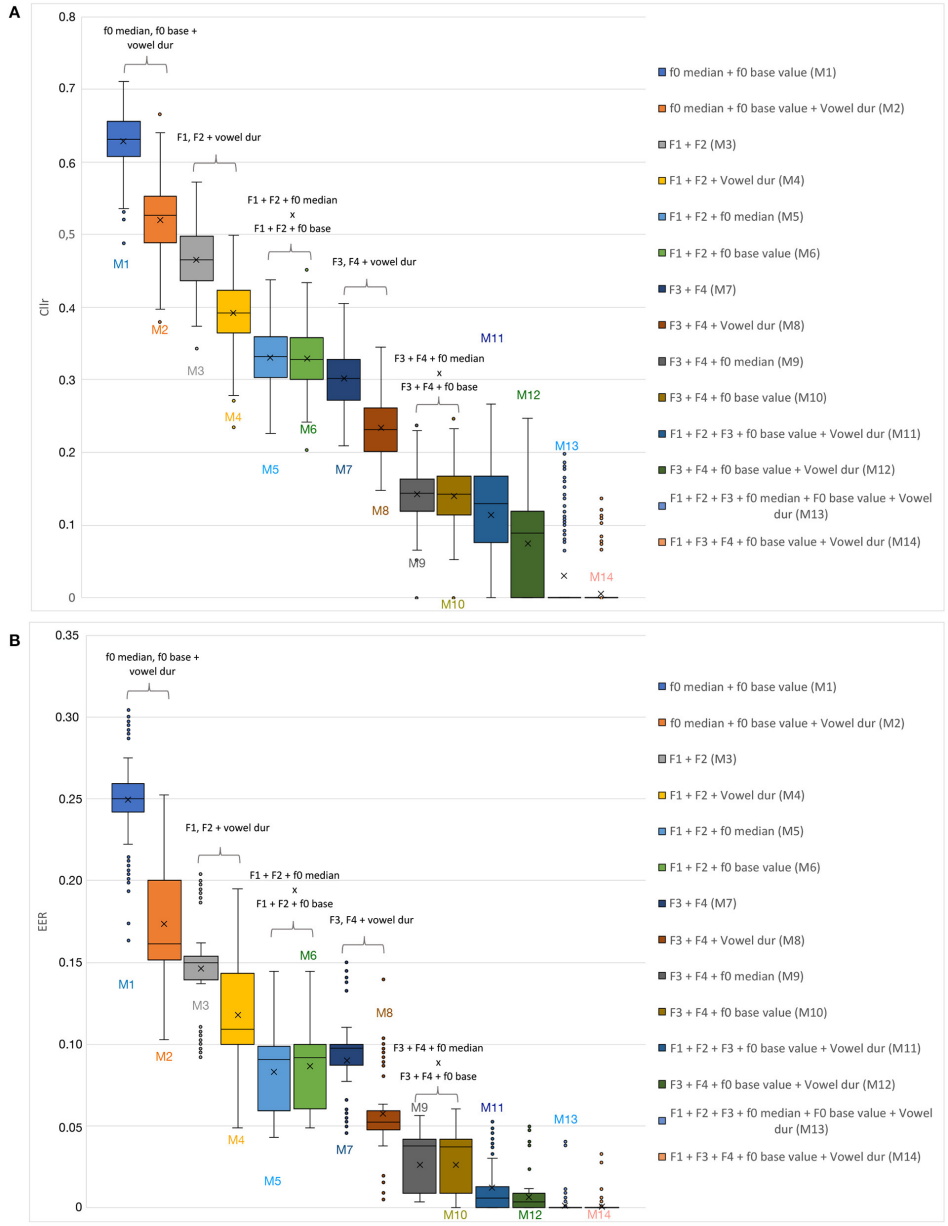
*Cllr* **(A)** and *EER* **(B)** cumulative values as a function of the combination of different acoustic-phonetic parameters for mismatched speaking styles (interview vs. dialogue).

In Figure 3, *Cllr* and *EER* values are depicted in the form of boxplots for all testing conditions, including speaker comparisons involving mismatched speaking styles, i.e., dialogue vs. interview. Finally, in Figure 4, *Cllr* and *EER* values are presented while considering the combination of some of the acoustic-phonetic parameters presented in Table 3 in increasing order of system performance, i.e., from lower to higher, with speaker comparisons involving mismatched speaking styles.

Furthermore, it must be acknowledged that the combinations of parameters presented in Table 3 and Figure 4 are not meant to be comprehensive, but to serve the purpose of the present analysis. It is also important to bear in mind that the selection of acoustic estimates to compose a statistical model in a forensic speaker comparison setting often comes down to the availability and suitability of parameters. Therefore, testing different models, even the least explanatory ones, is relevant.

### 3.1. Speaker comparisons within speaking styles

By inspecting the evolution of *Cllr* and *EER* values in Figure 1 as a function of different data configurations and acoustic parameters it does appear that data configuration seemed to have an effect on the calculations. Such an effect was found to be larger for some parameters in comparison to others. For instance, this is the case when comparing f0 base value in relation to other acoustic-phonetic parameters within the same speaking style.

However, despite the variability observed for *Cllr* and *EER* values in Figure 1, a clear trend is suggested concerning the regions in which the curves occupy in the graphs. As can be noted, from all parameters examined, speech tempo parameters, i.e., speech and articulation rates, were the curves depicting the highest *Cllr* and *EER* values when assessed in isolation for both speaking styles assessed.

Such a pattern is also evident when comparing the parameters *Cllr* and *EER* boxplots in Figure 2, where considerably higher median, arithmetic mean, maximum, and minimum *Cllr* and *EER* values were observed for the speech temporal category. Such a finding agrees with the assumption of speech tempo as being less speaker-discriminating when compared with the other phonetic estimates.

Conversely, as can be seen in Table 2 and Figure 2, spectral parameters, i.e., F3 and F4, were the ones depicting the lowest *Cllr* and *EER* values, which is compatible with their relatively higher speaker discriminatory potential. Moreover, within this category, F3 presented the lowest arithmetic mean, median, and minimum *Cllr* values for both speaking styles assessed. Among spectral parameters, F2 was the one depicting the weakest performance, being outperformed by melodic parameters in both speaking styles (see Table 2).

Furthermore, as can be noted by looking at Figure 2, melodic parameters came after spectral parameters in terms of their speaker discriminatory power. Although *f* 0 median performed slightly better in some cases, *f* 0 base value depicted the highest stability across all parameters within the same speaking style, displaying the lowest variability in *Cllr* and *EER* as a function of data configuration.

Finally, a trend that can be noted when inspecting Figure 2 is, with the exception of speech temporal parameters, a slightly better discriminatory performance was observed within the dialogue data in comparison to the interview data. This was the case when comparing Cllr and EER values across both speaking styles.

### 3.2. Speaker comparisons in mismatched speaking styles

A noteworthy observation regarding the assessment of individual acoustic-phonetic parameters in mismatched speaking styles is that the manner of speaking of a subject in specific and distinct communicative settings appears to affect the overall speaker discriminatory performance. As can be seen in Table 2 for the comparison between dialogue vs. interview, an increase in *Cllr* and *EER* average scores was observed for all parameters in relation to *Cllr* and *EER* scores obtained for the analysis based on the same speaking style. This overall weaker performance can be rationally explained, to a great extent, by an increase in intra-speaker variability, inflating the uncertainty regarding the “same” or “different” origins of the materials being compared.

However, as can be observed in Table 3 and Figure 4, a multidimensional analysis based on a range of sources of acoustic-phonetic information about a speaker seems to be of great importance when performing speaker comparisons in a mismatched speaking style condition. Combining different acoustic-phonetic parameters led to improvements in discrimination; the magnitude of improvement in discriminatory power varied as a function of the parameters being combined.

An interesting observation regarding Figure 4 is the fact that, although speech temporal parameters depicted the weakest performance when assessed in isolation, the inclusion of at least one speech temporal estimate tended to improve the overall performance, reducing the *Cllr* and *EER* scores. This was the case when combining melodic, and spectral measures with vowel duration, for instance (see *Model* 3 vs. 4). Moreover, the level of the combined discriminatory power was even greater for spectral in relation to melodic estimates.

It is worth noting that in certain cases, the combination of spectral and melodic parameters yielded better results than combining either melodic or spectral parameters with vowel duration., shifting the overall *Cllr* and *EER* scores downwards toward lower values (see *Models* M5 and M6 in Figure 4). This particularly applies to the combination of higher formant frequencies (i.e., F3 and F4) and melodic estimates (f0 base value or median), see *Models* M9 and M10 in Figure 4.

However, the addition of at least one speech temporal parameter (i.e., vowel duration) to a melodic and spectral-based model increased the model's discriminatory power (see *Model* M11 vs. M12 in Figure 4). That was not without a cost: a higher variability in *Cllr* scores. In order words, the magnitude of improvement in discriminatory power when including speech tempo parameters in the model was less substantial than when only spectral and melodic parameters were combined. However, the contribution of adding a speech tempo estimate to the whole model was noticeable, mainly when considering average *Cllr* and *EER* values between *Models* M11 and M12.

Finally, as can be noted by visually inspecting Figure 4, the best-performing models were the ones that included at least one or two parameters from different phonetic classes. Moreover, when comparing the two last *Models* in Figure 4, one observes that the inclusion or exclusion of one more melodic parameter, e.g., *f* 0 median in Model M13, resulted in a non-substantial change. The numerical detail of the results is presented in Table 3.

The reader should also be reminded that, although *Cllr* and *EER* average scores tend to approach zero in *Models* M13 and M14, in realistic forensic speaker comparison settings- often involving poor-quality recordings and a limited number of speech samples, the same outcome should not be expected. In the present analysis, only high-quality recordings have been used, which contained a relatively large amount of data.

A final observation that can be drawn from Table 3 regards the fact that, in some cases, the combination of estimates from the same phonetic class did not yield an as expressive discriminatory improvement as the combination of parameters from different acoustic-phonetic classes. This applies mainly to the melodic parameters tested. For instance, the combination of f0 median or base value plus vowel duration outperformed the combination of the two melodic parameters. A counterexample is the combination of spectral parameters. A model composed of only F1, F2, and F3, performed just as well (or slightly better) than the combination of F1, F2 plus a melodic estimate, suggesting the combination of only spectral parameters as an exception to this trend. Such a finding requires further exploration.

## 4. Discussion

This study set out to assess and compare the speaker discriminatory power of different acoustic-phonetic parameters deriving from three phonetic classes, namely, spectral, melodic, and temporal parameters. For that purpose, spontaneous dialogues between familiar speakers were analyzed. The main outcomes are discussed in the following.

### 4.1. On the comparison of different acoustic parameters

Previous studies have already acknowledged the relatively good discriminatory power of formant frequencies, especially higher formant frequencies, e.g., F3 and F4, cf. Loakes (2003), Gold et al. (2013), Cao and Dellwo (2019), and Cavalcanti et al. (2021a), as well as some centrality and base value measures of the speaking fundamental frequency, cf. Kinoshita et al. (2009), Silva et al. (2016), and Cavalcanti et al. (2021b).

Furthermore, despite the recognized higher resistance of speech timing estimates to different sources of audio degradation (e.g., external noise and the telephone bandwidth), previous studies have already pointed out their lower discriminatory capacity in relation to other acoustic-phonetic parameters when assessed in isolation (Künzel, 1997; Hughes et al., 2013; Lennon et al., 2019). Therefore, it is natural that one may question why this appears to be the case and what underlines such a discriminatory *asymmetry*.

A possible explanation why a lower level of speaker discriminatory power would apply to the speech tempo class in comparison with other phonetic classes may find ground on the premises of the H&H theory (Lindblom, 1990).

According to the H&H theory proposed by Lindblom (1990), speech production can be understood on the basis of an adaptive organization shaped by general biological processes. In this perspective, speakers are believed to adjust their speech performance according to communicative and situational demands, responding to the interplay between production-oriented factors and output-oriented constraints. Consequently, the influence of these factors should be expected to affect production along a continuum between hyper- and hypo-speech to obtain what the author calls “sufficient discriminability”. Moreover, according to the author, these adjustments or adaptations would reflect the speaker's awareness of the listener's ability to access information sources independent of the input (speech signal) and his judgment of the short-term demands for explicit information contained in the signal.

A possible reason for the observed lower inter-speaker discriminatory power may reside in constraints and demands imposed by the communication process itself. The level of variation admitted by any of the aforementioned classes, i.e., spectral, melodic, and temporal, is assumed to be regulated by the combination of intrinsic and extrinsic forces. These forces are expected to reverberate on the level of speaker separability of different acoustic parameters.

From this perspective, the level of variation observed in different parameters of speech production is far from a random or unregulated process; on the contrary, it seems conditioned to the implications it can bear on the communication process. Thus, the selection of potential estimates for speaker comparison ends should also consider such intrinsic and extrinsic factors underlying the phonetic variability.

Several studies support the observation that neural activity phase-locks to rhythm, e.g., Luo and Poeppel (2007), Doelling and Poeppel (2015), Ding et al. (2017), and Harding et al. (2019). In a literature review by Poeppel and Assaneo (2020), the authors explored studies with what they call the “temporal mesoscale” of speech, with particular attention to regularities in the envelope of the acoustic signal that correlate with syllabic information. It has been observed that the temporal structure of speech at this scale is remarkably stable across languages. As argued by the authors, this rhythmicity is required by the processes underlying the construction of intelligible speech.

The relevance of the outcomes of the referred studies in interpreting the present findings is that they seem to concomitantly signal the limits of variability expected for the rate of speech, suggesting an intertwined relation between production and perception. In that sense, although speakers do tend to vary in their temporal speech patterns, the magnitude of this variation may be seen as under production-oriented and output-oriented constraints, driven by demands of production efficiency on the one hand and comprehensibility on the other, cf. Lindblom (1990).

It should also be acknowledged that, as previous studies suggest, cf. Heuvel (1996) and Cavalcanti et al. (2022), an uneven level of speaker specificity appears to be present within the speech temporal domain itself. In Cavalcanti et al. (2022), for instance, it was observed that macro speech timing parameters (e.g., speech and articulation rates) appeared to contain more speaker-specific information in relation to micro speech timing parameters (e.g., vowel and syllable duration). According to the arguments presented in that paper, a low explanatory potential of the speaker identity regarding micro-temporal parameters may suggest those units as under a higher level of linguistic control. As such, variations in the fine temporal scale of speech would likely be better accounted for by linguistic/rhythmic constraints, whereas the effects of individual variation in speech timing would be more expressive in larger temporal windows.

In the present study, a decision was made to perform an analysis that is mainly based on the same phonetic unit: the vowel. The only two exceptions are the silent pauses and V-V unit duration parameters. However, when testing the combined discriminatory power of different acoustic-phonetic parameters, only vowel segments were chosen. In this regard, narrowing the acoustic analysis down to the same phonetic level may shed some light on the true nature of the speaker discriminatory power asymmetry reported here, tackling the issue of the uneven number of observations of parameters based on different measurement windows.

### 4.2. On the orthogonality of acoustic-phonetic parameters

As reported previously, combining estimates from the same phonetic class, such as melodic parameters, did not always improve discriminatory ability. However, when combining estimates from different phonetic classes, a better discriminatory performance tended to be observed. Overall, the most effective models typically included one or two parameters from different acoustic-phonetic classes.

It is worth remarking that previous studies have already reported the properties of systems composed of multiple parameters outperforming single parameter-based systems, as in Wang et al. (2022). Excluding some exceptions, the authors observed in that study that system validity and stability tend to improve when more acoustic features are involved. Systems with more acoustic features tended to shift toward lower Cllr values. Moreover, when intra-speaker likelihood ratio examinations were carried out, more accurate performances for multiple-feature systems were observed. However, the combination of multiple acoustic features also presented some consequences on the overall system stability. It is noteworthy to mention that systems that combine all available acoustic-phonetic features are not necessarily the most suitable ones. An overall consideration of the parameters being combined is still necessary.

In the present study, a plausible explanatory factor regarding the better performance of the system concerning the combination of different acoustic-phonetic parameters may reside in a theoretical facet of the analysis: the level of orthogonality of the acoustic-phonetic parameters incorporated into a statistical model. Here, orthogonality can be understood as the level of non-correlation that may be observed for different acoustic features and their degree of covariance.

As can be anticipated, acoustic-phonetic parameters that are under a lower orthogonal relation—as the relation between speech and articulation rates, and *f* 0 median and *f* 0 baseline)—tend to co-vary. Conversely, less inter-dependent parameters tend to contribute differently to the overall statistical model being tested, given their higher orthogonal association (i.e., higher degree of independence). Such logic may explain why the combination of *f* 0 base value and vowel duration, or *f* 0 median and vowel duration resulted in better discriminatory performance when compared to the combinations between *f* 0 base value and *f* 0 median, for instance (see Table 3). It was also noted that combining F1 and F2 to either *f* 0 base value or *f* 0 median did not result in an expressive change in system performance.

An exception was, of course, the combination of spectral estimates, e.g., F3 and F4, which resulted in an expressive reduction of *Cllr/EER* values and, therefore, in a markedly better system performance. The same principle may still account for such an exception. The level of covariance assumed for F3 and F4 can be considered lower than expected for centrality *f* 0 parameters and, consequently, their degree of covariance. In this sense, the combination of F3 with F4 adds some level of information “novelty” that helped improve discriminatory performance. In other terms, when constructing a statistical model based on acoustic features, one may ask what level of “newness” an acoustic feature add to the general model that is not already there.

Such a rationale may be at least one of the possible factors accounting for the difference in performance between different speaker-contrasting models. Other factors regard the intrinsic discriminatory potential of the acoustic-phonetic parameters themselves and their robustness.

## 5. Conclusions

The outcomes of the present study suggest the existence of a speaker discriminatory power *asymmetry* concerning different acoustic-phonetic parameters, in which speech tempo measures presented a lower discriminatory power compared to melodic and spectral parameters. Such a finding agrees with previous reports and bears important implications for forensic phonetics, suggesting that the parameters usually most robust to the effect of degradation due to poor audio quality, such as temporal estimates, are not necessarily the most discriminatory for forensic speaker comparison applications.

However, it must be noted that, when assessed in combination with other parameters, speech temporal estimates helped improve the system's overall performance, reducing *Cllr* and *EER* values in a mismatched speaking style testing condition. Furthermore, the best discriminatory performance was observed for models which combine parameters from different acoustic-phonetic classes, including melodic, spectral, and temporal parameters.

Finally, the results also signaled that *data sampling* appears to be of crucial relevance for the reliability of the results, given the observed variability of *Cllr* and *EER* values as a function of data selection. Future studies should look at the validity of the present results when comparisons of different speaking styles are made.

## Data availability statement

The datasets presented in this study can be found in online repositories. The names of the repository/repositories and accession number(s) can be found below: https://doi.org/10.6084/m9.figshare.21571866.v1.

## Ethics statement

The studies involving human participants were reviewed and approved by Campinas State University (UNICAMP), protocol 95127418.7.0000.8142. The patients/participants provided their written informed consent to participate in this study.

## Author contributions

JC, AE, and PB contributed to the conception, design of the study, and wrote sections of the manuscript. JC organized the database, performed the statistical analysis, and wrote the first draft of the manuscript. All authors contributed to the manuscript revision, read, and approved the submitted version.
